# RIM-DB: a taxonomic framework for community structure analysis of methanogenic archaea from the rumen and other intestinal environments

**DOI:** 10.7717/peerj.494

**Published:** 2014-08-05

**Authors:** Henning Seedorf, Sandra Kittelmann, Gemma Henderson, Peter H. Janssen

**Affiliations:** AgResearch, Grasslands Research Centre, Palmerston North, New Zealand

**Keywords:** Methanogen, Archaea, Taxonomy, Rumen, Intestinal microbiota, Reference database

## Abstract

Methane is formed by methanogenic archaea in the rumen as one of the end products of feed fermentation in the ruminant digestive tract. To develop strategies to mitigate anthropogenic methane emissions due to ruminant farming, and to understand rumen microbial differences in animal feed conversion efficiency, it is essential that methanogens can be identified and taxonomically classified with high accuracy. Currently available taxonomic frameworks offer only limited resolution beyond the genus level for taxonomic assignments of sequence data stemming from high throughput sequencing technologies. Therefore, we have developed a QIIME-compatible database (DB) designed for species-level taxonomic assignment of 16S rRNA gene amplicon data targeting methanogenic archaea from the rumen, and from animal and human intestinal tracts. Called RIM-DB (**R**umen and **I**ntestinal **M**ethanogen-**DB**), it contains a set of 2,379 almost full-length chimera-checked 16S rRNA gene sequences, including 20 previously unpublished sequences from isolates from three different orders. The taxonomy encompasses the recently-proposed seventh order of methanogens, the Methanomassiliicoccales, and allows differentiation between defined groups within this order. Sequence reads from rumen contents from a range of ruminant-diet combinations were taxonomically assigned using RIM-DB, Greengenes and SILVA. This comparison clearly showed that taxonomic assignments with RIM-DB resulted in the most detailed assignment, and only RIM-DB taxonomic assignments allowed methanogens to be distinguished taxonomically at the species level. RIM-DB complements the use of comprehensive databases such as Greengenes and SILVA for community structure analysis of methanogens from the rumen and other intestinal environments, and allows identification of target species for methane mitigation strategies.

## Introduction

Ruminants such as sheep and cattle are among the most numerous farmed animals, and enteric methane formation in their rumens contributes substantially to global greenhouse gas emissions (Yusuf et al., 2012), as well as being a conversion of part of the feed energy into a form unavailable to the ruminant animals. Methane is produced by methanogenic archaea, a phylogenetically diverse group of microorganisms (Janssen & Kirs, 2008). Depending on the species and substrate availability, methanogens can grow hydrogenotrophically (using hydrogen or formate), aceticlastically (using acetate) and/or methylotrophically (using methanol or other simple methyl-compounds) (Thauer, 1998). Rumen methanogens consume hydrogen, formate, and methyl-compounds that are among the products formed during the degradation and fermentation of ingested feed through the combined activities of bacteria, fungi, and protozoa. Aceticlastic methanogenesis does not appear to be a significant source of methane in the rumen and has only been observed in exceptional cases (Rowe et al., 1979). Methane cannot be used by the ruminant and it is lost to the atmosphere, mainly through eructation. To mitigate emissions of methane from ruminants into the atmosphere, interventions are being developed to reduce the number or activity of methanogenic organisms in the rumen (Buddle et al., 2011). Interventions like targeted vaccines and inhibitors are based on genome sequences of key methanogens (Leahy et al., 2010). In addition, tools based on DNA markers (Kittelmann et al., 2013) are being used to monitor the effects of experimental interventions or to uncover differences in microbial community structures in animals with different productivity traits, such as differences in feed conversion efficiency (Carberry et al., 2014; Zhou, Hernandez-Sanabria & Guan, 2009; Zhou, Hernandez-Sanabria & Guan, 2010). Therefore, the accurate identification and classification of rumen methanogens is an important step, whether to identify target methanogens, for subsequent community analysis during the development and testing of mitigation technologies, or to uncover details of rumen community differences in animals with different production characteristics.

Next generation sequencing technologies have revolutionised cultivation-independent methods of characterising microbial communities, and it has become feasible to sequence amplicons at unprecedented depth and from large numbers of samples. Having overcome the hurdle of generating large enough amounts of sequencing data to cover a large part of the diversity of the microorganisms present in a sample, the focus now needs to shift to another crucial aspect of community structure characterisation, namely the correct taxonomic assignment of sequencing reads. This step relies on the availability of high-quality reference sequences for the relevant environment. The rumen is known to contain methanogens from at least four different orders: Methanobacteriales, Methanomicrobiales, Methanosarcinales and Methanomassiliicoccales (Janssen & Kirs, 2008). The availability of reference 16S rRNA gene sequences for each order varies considerably and is particularly low for Methanomassiliicoccales, which have recently been proposed as a new order of methanogens (Iino et al., 2013) (for which the synonymous name ‘Methanoplasmatales’ has also been proposed (Paul et al., 2012)). Only six almost full-length sequences (>1,200 bp) from isolates or enrichment cultures are currently publicly available for Methanomassiliicoccales, making it difficult to obtain a comprehensive overview of this order and to define taxonomic groups. This limitation can partially be overcome by including almost full-length PCR-amplified sequences from cultivation-independent studies, especially older clone library-based investigations. The downside of this approach is that these sequences may potentially be of lower quality. Cultivation-independent studies rely on sequenced PCR products and may include artefacts, such as chimeras (Hugenholtz & Huber, 2003). The removal of such low quality sequence data from reference databases is therefore necessary to produce high quality and trustworthy taxonomic assignments and to reliably define taxonomic groups that do not yet have cultured representatives.

The aim of the work described here was to develop a taxonomic framework that improves the quality and accuracy of taxonomic assignment of rumen and intestinal methanogens. We therefore developed a specialised taxonomic framework, a ***R*** *umen and* ***I*** *ntestinal* ***M*** *ethanogen* ***D*** *ata* ***b*** *ase* (RIM-DB). RIM-DB includes 16S rRNA gene sequences from ruminal methanogens, and, to make it more widely useful, from various other intestinal environments where methanogens are known to be important hydrogen consumers, such as the human (Samuel & Gordon, 2006; Miller et al., 1982) and termite intestinal tract (Brune, 2014). The database contains long (>1,200 bp) 16S rRNA gene sequences that have been checked for potential chimeras. We believe that the use of specialised and/or refined taxonomic frameworks allows more detailed and accurate taxonomic assignments than other publicly available databases, such as Greengenes or SILVA, which are far more comprehensive and contain highly diverse sets of 16S rRNA sequences from many different environments (McDonald et al., 2012; Pruesse et al., 2007), but which, due to their size, are not curated to the same extent. We tested the newly developed RIM-DB by analysing the composition of the methanogen microbiota in the rumens of sheep and cattle in New Zealand and compared the results to those obtained using SILVA and Greengenes taxonomies.

## Materials and Methods

### Sequence selection

Archaeal 16S rRNA gene sequences were exported from SILVA version 111 (Pruesse et al., 2007) to develop a database that represents the seven orders of methanogens and the order Archaeoglobales. Initially, all sequences that fell into one of the five established orders (Methanobacteriales, Methanococcales, Methanomicrobiales, Methanopyrales, and Methanosarcinales (Bonin & Boone, 2006; Garcia, Ollivier & Whitman, 2006; Huber & Stetter, 2001; Kendall & Boone, 2006; Whitman & Jeanthon, 2006)) and into the two recently-proposed orders, Methanocellales and Methanomassiliicoccales (Sakai et al., 2008; Iino et al., 2013), were selected. Sequences from the thermophilic genera of the order Methanobacteriales were removed from this selection because they are not known to be major colonisers of the rumen or the intestinal tracts of animals or humans. Sequences of thermophilic methanogens belonging to other orders and the non-methanogenic order Archaeoglobales were retained in the dataset so that it included representatives of all methanogenic and closely-related orders. Twenty-eight sequences of methanogens not represented in the SILVA 111 database were added to the sequence database (Table S1). Another twenty sequences were made publicly available as part of this study and were also added to the database (Table S1). These sequences include three novel cultured representatives of the order Methanomassiliicoccales. All RIM-DB sequences were subjected to rigorous chimera checks using a BLAST-based chimera checking protocol and the UCHIME pipeline, which are both part of the QIIME package, version 1.5 (Caporaso et al., 2010; Edgar et al., 2011). Both methods were run in reference mode, using 16S rRNA gene sequences from methanogen type strains and the Greengenes database (release GG_05_13) as reference sets, respectively. Sequences identified as chimeric by the *parallel_identify_chimeric_seqs.py* script, using the options –d 4 and –n 2, were removed from the database. Questionable sequences that were identified by UCHIME were manually inspected by BLAST (Altschul et al., 1990) analysis of 5′ and 3′ ∼400 bp-long fragments and discarded when considered to be chimeric. Duplicate sequences were eliminated unless they were derived from isolates or stable cultures. Following these steps, 2,379 sequences remained for analysis, and these are provided as a qiime-compatible database file (File S1).

### Alignment, tree construction and taxonomic assignment

Sequences were aligned using the SINA aligner using default settings (Pruesse, Peplies & Glöckner, 2012), then imported into ARB and the alignment, where necessary, manually curated (Ludwig et al., 2004). Aligned sequences were exported in phylip format to construct phylogenetic trees using all available base positions. Maximum likelihood phylogenetic trees based on aligned archaeal 16S rRNA gene sequences were generated using RAxML version 7.0.3 (Stamatakis, 2006). Unless stated otherwise, the parameters “-m GTRGAMMA -# 500 -f a -x 2 -p 2” were used. Taxonomic strings were generated for each of the database sequences according to the naming scheme used in the Greengenes taxonomy, consisting of seven different taxonomic levels: k_kingdom; p_phylum; c_class; o_order; f_family; g_genus; s_species. Two different qiime-compatible taxonomy files (File S2 and File S3) are provided, both intended to be used in conjunction with the reference sequence database (File S1). The taxonomy file in File S2 contains an eighth field, “i_isolate”, which has been introduced to designate which sequences originate from isolates or cultures. This file contains the taxonomic strings used in the ARB database (File S4). In the taxonomy file in File S3, most sequences were manually binned into species-level clades to define an association of the majority of sequences to known species. This file is intended for taxonomic assignment of sequences to designated taxa, for example when analysing large datasets of archaeal 16S rRNA gene sequences originating from the rumen or other gastrointestinal systems. Phylogenetic trees shown in the paper are also included in the ARB-database that is provided (File S4).

### Positional coverage and variability

Sequence coverage at each alignment position was calculated to determine if RIM-DB was suitable for taxonomic assignments of amplicon reads obtained from different regions of the archaeal 16S rRNA gene. Variability of base composition for sequences in RIM-DB was analysed in R version 3.0 (R Development Core Team, 2005) using the seqinR package (Charif & Lobry, 2007), and results were plotted using the ggplot2 library (Wickham, 2009). Shannon index values for each alignment position were calculated using the frequencies of bases A, T (U and T were used synonymously), G, C and blank positions (Shannon, 1948). Variable regions for the analysis were selected as described previously (Hartmann et al., 2010; Kim, Morrison & Yu, 2011).

### Definition of groups in the different orders of methanogens

Sequences of poorly-resolved clades were binned into defined groups to improve the accuracy and detail of taxonomic assignments as follows. All groupings of sequences was done in ARB (Ludwig et al., 2004). The groupings were primarily based on sequence identity by identifying sequences within a chosen identity cut-off (mostly 95%–97%) for a selected sequence and strong bootstrap support (≥70%) for any defined group.

### Benchmarking against other databases

Benchmarking was performed on isolate sequences and on an amplicon test dataset. Twenty-four sequences of rumen and intestinal methanogens were selected for benchmarking with isolate sequences (see Table S2). The selected sequences were either exported from SILVA or are published as part of this study (see Table S2 for isolates). Analysis was performed on long length (>1,000 bp) sequences and on sequences of the V6–V8 variable regions of the 16S rRNA gene. Taxonomic assignment of sequences was carried out using the *parallel_assign_taxonomy_blast.py* script in QIIME, version 1.5. The three different reference databases used for taxonomic assignments of sequences were RIM-DB (File S1 and File S3), SILVA (release 111, Pruesse et al., 2007) and Greengenes (release GG_13_05, McDonald et al., 2012). QIIME-compatible SILVA and Greengenes databases were downloaded from http://qiime.wordpress.com. Specific options/files used for taxonomic assignments with SILVA were: –id_to_taxonomy Silva_111_taxa_map_full.txt and –blast_db Silva_111_full_unique.fasta; and with Greengenes: –id_to_taxonomy gg_13_5_taxonomy and -blast_db gg_13_5.fasta. Abundance tables were generated and only OTUs with a mean minimum relative abundance of 1% across all samples were retained.

A test set of amplicon sequence data was generated by combining the following sequence datasets (for accession numbers see Table S4). These datasets contain partial 16S rRNA gene sequences covering nucleotide positions 935–1,385 (*Escherichia coli* 16S rRNA nucleotide numbering (Brosius et al., 1978)). Sequence data were processed using the QIIME package, version 1.5 (Caporaso et al., 2010). Reads were quality filtered and assigned to the corresponding sample by barcodes using the QIIME *split_library.py* script. Only reads with average quality scores >25 were included in the analysis. The resulting fna-files from all experiments were concatenated and denoised using combined flowgram-files, using the *denoise_wrapper.py* script with default settings (Reeder & Knight, 2010). The output was subjected to the *inflate_denoiser_output.py* script (*default settings*). *Denoised sequence reads were chimera-checked with the* QIIME script *parallel_identify_chimeric_seqs.py*, using the parameters –d 4 and –n 2, and using RIM-DB as the reference database. The chimeric sequences that were identified were removed from the dataset using the QIIME *filter_fasta.py* script. Subsequently, the denoised and chimera-checked dataset was processed with the QIIME pipeline. Sequences were clustered into operational taxonomic units (OTUs) used the default clustering method UCLUST (Edgar, 2010) *with* a sequence similarity cut-off of 99% (*pick_otus.py* option: -s 0.99). Abundance tables were generated and only OTUs with a mean minimum relative abundance of 1% across all samples were retained. Taxonomic assignment of representative sequences was carried out as described for the isolate sequences.

## Results and Discussion

Taxonomic frameworks are essential tools for describing the composition of microbial community structures. This study had two main aims. We wanted to develop a taxonomic framework for rumen methanogens and methanogens residing in other intestinal habitats based on long (>1,200 bp) 16S rRNA gene sequences that resolves taxonomic groups that are currently unresolved by taxonomies such as Greengenes or SILVA. We achieved this by better defining taxonomic groups (corresponding to the genus and species levels) within the genera *Methanobrevibacter* and *Methanosphaera* and the order Methanomassiliicoccales to develop RIM-DB. We then wanted to test the utility of this framework to provide more detailed taxonomic assignments for shorter sequence data (covering the V6–V8 region) obtained from rumen microbial communities.

### Construction of the database

**Figure 1:**
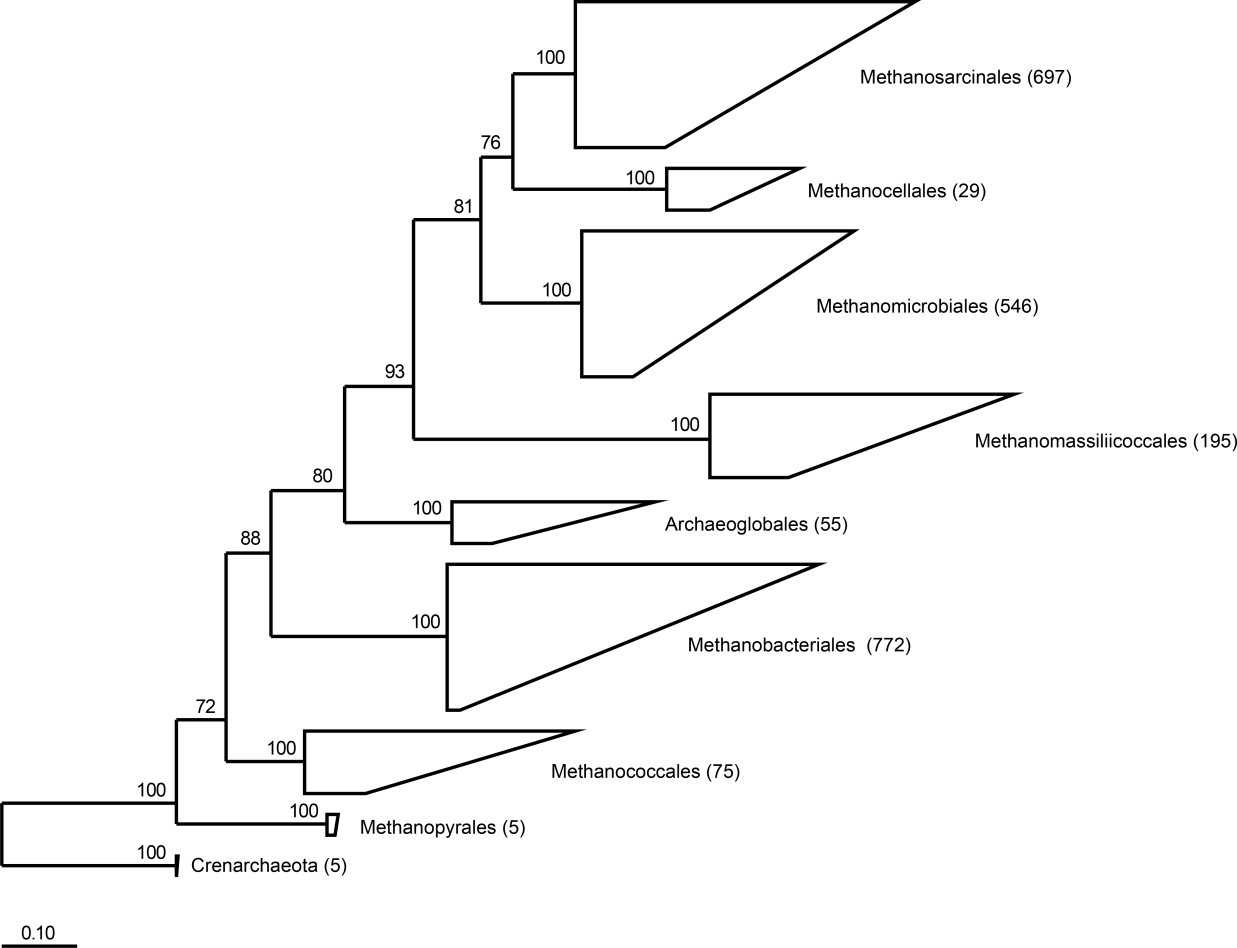
Phylogeny of the seven orders of methanogens based on near full length 16S rRNA gene sequences. Numbers in parentheses after the order names indicate the number of sequences that are present in RIM-DB for each of these orders. Detailed overviews of the orders Methanobacteriales and Methanomassiliicoccales are shown in Figs. S1 and S2, respectively. The tree was re-sampled 250 times and only bootstrap values ≥70% are shown. The dendrogram was rooted with five Crenarchaeota sequences. The scale bar indicates 0.10 inferred nucleotide substitutions per position.

RIM-DB contains a subset of currently available archaeal 16S rRNA gene sequences and is primarily designed for taxonomic assignment of methanogenic archaea from the rumen and other intestinal environments. A maximum likelihood-based phylogenetic analysis of 2,379 high-quality sequences indicated the existence of seven clearly-defined orders of methanogenic archaea (Fig. 1). Previous studies have shown that ruminants from different geographic locations around the world are dominated by members of only two of the seven orders, the Methanobacteriales and the Methanomassiliicoccales (Kittelmann et al., 2013; Jeyanathan et al., 2011; King et al., 2011; Wright, Ma & Obispo, 2008; Wright, Toovey & Pimm, 2006; Wright et al., 2004). Within the Methanobacteriales, the genera *Methanobrevibacter* and *Methanosphaera* have primarily been detected in the rumen; and both genera contain species that have also been found in a variety of other intestinal environments. Based on sequence similarity and bootstrap support, we combined database sequences into groups and developed a naming scheme for clades within the genera *Methanobrevibacter* and *Methanosphaera* and within the order Methanomassiliicoccales (Fig. S1 and Fig. S2).

The high degree of 16S rRNA gene sequence similarity between some *Methanobrevibacter* spp. is reflected by the grouping of sequences from a number of species into one clade that cannot be separated further, which prevents a more detailed taxonomic assignment of some sequencing reads. Within the genus *Methanobrevibacter*, *Mbb. ruminantium* and *Mbb. gottschalkii* were each defined as clades that include some other formally described species, specifically *Mbb. ollyeae*, *Mbb. millerae*, and *Mbb. thaueri* (Miller & Lin, 2002; Rea et al., 2007). The latter three species have been defined based on DNA hybridisation and other phenotypic characteristics, but sequence divergence between the 16S rRNA gene sequences of these strains is in some cases <2% (Rea et al., 2007). In RIM-DB, the type strains of *Mbb. millerae*, *Mbb. olleyae* and *Mbb. thaueri* have been assigned their given species names, but due to the high sequence similarity, *Mbb. thaueri* and *Mbb. millerae* are placed in the *Mbb. gottschalkii* clade, while *Mbb. ollyeae* is placed in the *Mbb. ruminantium* clade as previously suggested by Janssen & Kirs (2008). The low degree of sequence difference means that, based on 16S rRNA gene sequences, these *Methanobrevibacter* spp. (five named species plus further potential unrecognised species) cannot be further broken down below the level of these two clades that are typified by the species *Mbb. ruminantium* and *Mbb. gottschalkii*. Outside these two clades, sequences from other named *Methanobrevibacter* spp. and good-quality sequences can be defined to the species level, in some cases forming coherent groups that probably represent new species of *Methanobrevibacter* (Fig. S1). Among these are the already recognised *Mbb. smithii*, *Mbb. woesii*, and *Mbb. wolinii*, and potentially new species from rabbits, rats, and termites.

The genus *Methanosphaera* comprises methylotrophic methanogens within the order Methanobacteriales, and some of the characterised members are known to be restricted to methanol and hydrogen as substrates for methane formation (Fricke et al., 2006; Miller & Wolin, 1985). *Msp. stadtmanae* and *Msp. cuniculi* are currently the only formally-described species of this genus (Miller & Wolin, 1985; Biavati, Vasta & Ferry, 1988). The previously unpublished isolate sequences of *Methanosphaera* sp. ISO3-F5 (J Jeyamalar, GE Naylor, G Henderson, RS Ronimus, PH Janssen, 2010, unpublished data) and *Methanosphaera* sp. A4 (CC Kim, GE Naylor, R Ronimus, G Henderson, PH Janssen, 2012, unpublished data) indicate that at least two additional species may exist. These two isolates have less than 98% sequence identity to other described *Methanosphaera* species, but await formal description to confirm their species status. A fifth species level group was defined based on sequence divergence to other isolates and strong bootstrap support for the group (≥70%; *Methanosphaera* sp. Group5) (Fig. S1). No isolate sequences are available for this group to date. Based on the 16S rRNA gene, this group is most similar to *Methanosphaera* sp. ISO3-F5 (97.1% sequence similarity).

Using RIM-DB, 17 of 195 Methanomassiliicoccales sequences were assigned to the genus *Methanomassiliicoccus*, while the remaining 178 sequences were distributed into 12 newly-defined groups (Fig. S2). Most of these groups are strongly supported by high bootstrap values. Group 7 is the only group that contains only a single sequence in RIM-DB, which represents the enrichment culture *Methanogranum caenicola* Kjm51a (Iino et al., 2013), and no other (>1,200 bp) sequences appear to be currently available. Minimum sequence identities between some groups are higher than 95%, indicating that not all groups may represent potential new genera. In addition, sequence identities of less than 88% between the described *Methanomassiliicoccus* species and the previously unpublished cultures ISO4-G1, ISO4-G11, ISO4-H5 indicate a wide sequence divergence of species/genera within this order. It could also indicate that the recently proposed family Methanomassiliicoccaceae is not the only family in this order (Iino et al., 2013). The confirmation and proposal of a second family awaits formal description of some of its representative members. In the meantime, all Methanomassiliicoccales sequences are placed in the family Methanomassiliicoccaceae in the current version of this database. It has to be emphasised that these groupings (and group names) do not represent official nomenclature and that they await confirmation by successful isolation and description of representative isolates. They can, however, be used to provide a better definition of subgroups within the order.

Where applicable, sequences have also been distributed into groups of gastrointestinal methanogens from the genera *Methanobacterium*, *Methanimicrococcus* and *Methanosarcina*. The low abundance of these groups in our test dataset, and the lack of publicly-available datasets from intestinal samples that include significant numbers of sequences from these organism groups, currently prevent sufficient testing. However, their inclusion in the database will allow future identification of samples where these genera might be abundant, which could then provide more sequence data and justify further effort in delineating sub-groups.

### Analysis of sequence coverage for different variable regions

**Figure 2:**
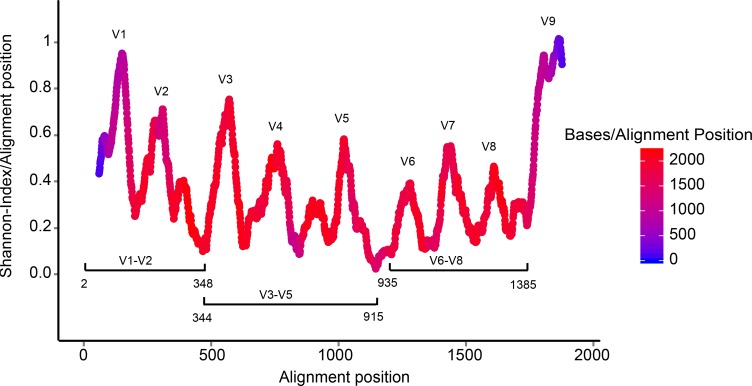
Coverage of the archaeal 16S rRNA gene by sequences included in RIM-DB. Sequence variability is expressed as Shannon-index for each alignment position using a 50-bp moving average. Sequence coverage per base is indicated by the heatmap and was calculated using a 50-bp moving average. Approximate positions of frequently targeted regions (V1–V2, V3–V5 and V6–V8) for amplicon sequencing are shown for orientation and nucleotide numbering corresponds to positions in the *Escherichia coli* 16S rRNA gene.

Currently available next generation sequencing methods target relatively short segments of the 16S rRNA gene (of up to approximately 500 bp in length). RIM-DB contains primarily sequences longer than 1,200 bp that cover large parts of the ∼1,540 bp long gene. Shorter sequences were only retained in the database if they originated from isolates or enrichment cultures. An analysis of the nucleotide position coverage by the database revealed that the coverage is even throughout the majority of the 16S rRNA gene (Fig. 2). This indicates that RIM-DB could also be suitable for the analysis of amplicon data that have been generated for variable regions other than the V6–V8 region, which was the target for amplicon sequencing of the samples included in the test dataset.

### Differences in taxonomy assignments using different databases

To compare the detail and accuracy of taxonomic assignment of sequences using RIM-DB in comparison to using Greengenes (release gg_13_5) or SILVA (release 111) databases, we analysed the taxonomic assignment of 24 different isolates and of a test dataset of amplicons.

**Figure 3:**
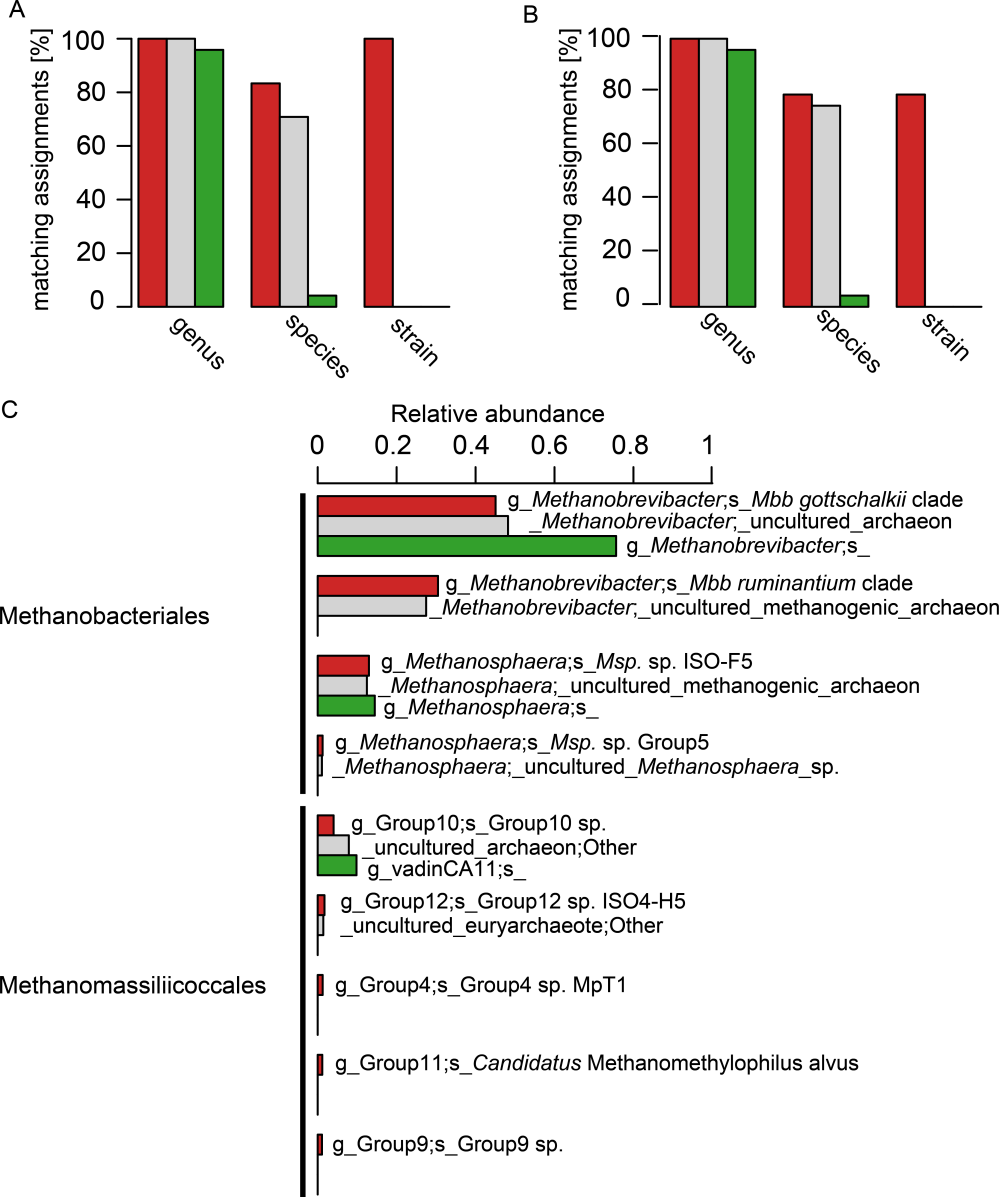
Comparison of taxonomic assignments using RIM-DB, SILVA, and Greengenes databases. Taxonomic assignments of (A) 24 long sequences from isolates and (B) of the V6–V8 region of the same 24 sequences were made using RIM-DB (red), SILVA (grey), and Greengenes (green) databases. Matching assignments with genus, species and strain names are given in percent for each of the different databases (see Table S2 for all assignments). (C) Taxonomic assignments of a large test dataset were made using the three different databases (see methods for details). These were summarised at the species level, and relative abundances of the most dominant groups were plotted according to the ranking of relative abundance of organisms groups assigned with RIM-DB. Differences in taxonomic assignments at the genus, family and order level have been omitted for simplicity (for a full taxonomic assignment see Table S3). Only OTUs with a relative abundance >1% are shown.

The 24 selected isolate sequences of rumen and intestinal methanogens spanned the orders Methanobacteriales, Methanomicrobiales, Methanosarcinales and Methanomassiliicoccales. Two versions of this sequence set were tested: the complete sequences and an extracted set of their V6–V8 regions. A comparison at the genus level revealed a high accuracy of taxonomic assignment, and little difference between assignments with the three different databases (Figs. 3A and 3B). At the species level, however, taxonomic assignments differed considerably. RIM-DB and SILVA performed well at species level assignment of these sequences, and assigned most long length and V6–V8 region sequences correctly (Figs. 3A and 3B). Resolution of taxonomic assignment was limited for highly similar sequences, such as *Methanobrevibacter boviskoreani* and *Methanobrevibacter* sp. AbM4, which share more than 99% sequence identity. Some sequences, e.g., of *Methanobrevibacter olleyae*, were assigned correctly by SILVA but not by RIM-DB, because *Mbb. olleyae* sequences and closely related *Mbb. ruminantium* sequences have been combined to a species level “*Mbb. ruminantium* clade” in RIM-DB. The “i_isolate” field in RIM-DB (File S2) did allow identification of all 24 isolates correctly when analysing long length sequences, and 19 isolates when analysing V6–V8 region sequences. It needs to be emphasized that sequencing errors and limited sequence divergence may make it difficult to classify sequences from environmental sequence data beyond the species level. Because of this, we developed a more conservative taxonomy in which some difficult-to-delineate sequences are grouped into clades. We therefore recommend that the taxonomic assignment of large datasets of short archaeal 16S rRNA gene sequences originating from the rumen or other gastrointestinal systems be performed using the more conservative taxonomy (File S3) in conjunction with the sequence database (File S1). Taxonomic assignments at the isolate level and at high similarity thresholds (>99%) are more useful for the analyses of samples of known composition, such as mock-communities, and could use the more detailed taxonomy file (File S4). Taxonomic assignment using the Greengenes database resulted in one of 24 sequences being correctly assigned at the species level (both for long length 16S rRNA sequences and for sequences spanning the V6–V8 region). Twenty-two sequences were not assigned at the species level, and one sequence was assigned erroneously (Figs. 3A and 3B, for full taxonomic assignments see Table S2).

This approach of testing and comparing taxonomic assignment with different databases is biased towards isolates present in the databases. For example, only few sequences of described Methanomassiliicoccales species are available. We therefore also analysed 520,563 sequencing reads of a test dataset of amplicons of V6–V8 regions of archaeal 16S rRNA gene sequences (Fig. 3C). All datasets were generated from sheep and cattle on different feeds, from different geographic locations within New Zealand, and of varying age (see Table S4 for details). The sequences in the datasets were combined, denoised, chimera-checked, clustered into 322 OTUs based on a clustering cut-off of 99%, and taxonomically assigned using the three different databases. The total relative abundances for each of the three most abundant organism groups (*Methanobrevibacter*, *Methanosphaera*, Methanomassiliicoccales) were similar, and, in general, differed by less than five percent between assignments with different taxonomies (see Table S3 for details). However, the detail of taxonomic assignment varied, depending upon which database was used. RIM-DB was able to distinguish between distinct *Methanobrevibacter* clades and assigned them as such (i.e., *Mbb. gottschalkii* clade, *Mbb. ruminantium* clade), whereas SILVA and Greengenes did not provide specific taxonomic assignments beyond the genus *Methanobrevibacter* genus level (Fig. 3C). SILVA did assign the *Methanobrevibacter*-associated reads to two groups, but there were not given any clear taxonomic designations to indicate what they might be. Greengenes assigned all *Methanobrevibacter*-associated reads to one undifferentiated *Methanobrevibacter* group (Fig. 3). Obtaining more detailed taxonomic assignments is important for characterising methanogen communities, because *Methanobrevibacter* spp. are among the most dominant methanogens in intestinal tracts of many mammals and termites (Janssen & Kirs, 2008; Dridi et al., 2009; Deevong et al., 2004; Leadbetter, Crosby & Breznak, 1998; Leadbetter & Breznak, 1996), and types of species may vary depending on host or host-tissue/organ (Janssen & Kirs, 2008; Dridi et al., 2009; Leadbetter, Crosby & Breznak, 1998; Leadbetter & Breznak, 1996; Miller & Wolin, 1982). In addition, the abundance of different clades of *Methanobrevibacter* spp. may be correlated with the abundance of certain bacterial groups (Kittelmann et al., 2013). Strains of the *Mbb. gottschalkii* and *Mbb. ruminantium* clades have been described as dominant species in the rumen, while *Mbb*. *curvatus*, *Mbb. filiformis*, and *Mbb. cuticularis* were isolated from termite guts and *Mbb. smithii* is from human faeces (Miller et al., 1982; Leadbetter, Crosby & Breznak, 1998; Leadbetter & Breznak, 1996). Other *Methanobrevibacter* strains, such as the less dominant rumen methanogen *Methanobrevibacter* sp. AbM4, a member of *Mbb. wolinii*, have been found to be involved in differences in feed efficiencies in ruminants (Zhou, Hernandez-Sanabria & Guan, 2009; Zhou, Hernandez-Sanabria & Guan, 2010). These examples underline the importance of detecting intra-genus differences for the analysis of amplicon data.

*Methanosphaera* sequences in the test sequence reads with RIM-DB were primarily assigned to *Methanosphaera* sp. ISO3-F5 (13% ± 7.7% [mean ± standard deviation]), a potential species-level group containing an isolate from a sheep rumen. Using RIM-DB, a small percentage (1.2% ± 1.7%) of reads of the test data set were assigned to the *Methanosphaera* sp. Group5 within the genus that does not contain any isolates, indicating that some species of *Methanosphaera* in the rumen are as yet uncultured. Taxonomic assignment with SILVA and Greengenes were restricted to the genus-level assignment and did not distinguish between different species of *Methanosphaera* in the test dataset (see Table S3 for full assignments).

Accurate taxonomic assignments of Methanomassiliicoccales sequences are difficult, because this order has just recently been proposed and *Methanomassiliicoccus* is currently the only validly described genus of this diverse order (Dridi et al., 2012). While assignment with SILVA and Greengenes suggested only two and one taxonomic group(s) respectively, taxonomic assignment with RIM-DB revealed the presence of five different potential species-level groups within Methanomassiliicoccales in the test dataset (Fig. 3). Not unexpectedly, most of the assigned OTUs could be assigned to groups 10 and 12, which tend to contain sequences from the rumen. Paul et al. (2012) already showed that Methanomassiliicoccales 16S rRNA and *mcrA* gene sequences could potentially be distributed into environment specific clades. We have extended these groupings by distributing additional reference sequences into clades, some of which appear to be dominated by sequences associated with a specific habitat. For example, group 12 of Methanomassiliicoccales contains primarily sequences from rumen (Fig. S2). Future efforts in community profiling of various environments using RIM-DB will verify whether these groupings are indeed habitat-specific. Both phylogeny and taxonomy of the Methanomassiliicoccales are at an early stage, and further efforts of isolating and characterising new species are required to fully understand the diversity of this interesting order of methanogens.

## Conclusions

Taxonomic frameworks require constant updating to accommodate changes in nomenclature and to include new sequence information. RIM-DB incorporates recent advances, such as the newly proposed order Methanomassiliicoccales, for which little taxonomic information is available. RIM-DB can provide new and detailed insights into the composition of the rumen archaeal microbiota, and our testing shows that this specialised taxonomy could be useful to complement analyses made using larger and more general databases such as Greengenes and SILVA. Some specialised taxonomies already exist (for examples see Kittelmann et al., 2012; Santamaria et al., 2012), but developing and refining additional specialised taxonomies should be considered, as these could also contribute to the refinement of general and more comprehensive databases.

RIM-DB is intended for taxonomic assignment of next generation amplicon sequence data of rumen methanogens, but it also includes reference 16S rRNA gene sequences from other intestinal habitats, such as human faeces and the termite gut. There is currently a lack of suitable publicly-available amplicon datasets, which prevents sufficient testing of RIM-DB on sequence data from habitats other than the rumen, but RIM-DB may also be a valuable resource for the analysis of methanogenic community structures from other intestinal environments, including the human gut. Like other taxonomic frameworks, RIM-DB will require updating to include novel sequences of rumen and intestinal methanogens and to address changes in nomenclature. The most recent release of RIM-DB (including fasta-, taxonomy- and ARB-file) is available for download from www.globalrumencensus.org.nz.

## Supplemental Information

10.7717/peerj.494/supp-1 Figure S1 Phylogeny of Methanobacteriales based on the 16S rRNA gene and on sequences included in the taxonomic framework Abbreviations: *Mbm.* = *Methanobacterium*, *Mbb*. = *Methanobrevibacter*, *Msp*. = *Methanosphaera*. The tree was re-sampled 500 times and only bootstrap values ≥70% are shown. The dendrogram was rooted with five Crenarchaeota sequences. The scale bar indicates 0.10 inferred nucleotide substitutions per position.

10.7717/peerj.494/supp-2 Figure S2 Phylogeny of Methanomassiliicoccales based on 16S rRNA gene sequences included in the RIM-DB taxonomic framework. The tree was re-sampled 500 times and only bootstrap values ≥70% are shown. The dendrogram was rooted with 55 sequences from the Archaeoglobales. Available representative isolates/enrichment cultures for the groups are listed after the group name. Alternative group names (Group 2a = TC-2, Group 2b = CC-2, Group 4 contains CC-1, TC-1a, and TC-1b) have been given for some of the groups by Paul and colleagues (2012). The font colour used for the isolate names represents the habitat from which the strains were isolated or enriched: rumen (green), human (red), termite (brown), sludge (grey). Short isolate sequences were not included in the tree. The scale bar indicates 0.10 inferred nucleotide substitutions per position.

10.7717/peerj.494/supp-3 Table S1 New 16S rRNA gene sequence information Sequences that were added to RIM-DB and that are not included in SILVA 111.

10.7717/peerj.494/supp-4 Table S2 Taxonomic assignments of 16S rRNA gene sequences from isolates made using RIM-DB, SILVA, and Greengenes databases Shown are the taxonomic assignments of (A) long length isolate sequences and (B) of V6–V8 region sequences obtained using the three different databases.

10.7717/peerj.494/supp-5 Table S3 Species-level taxonomic assignment of test set sequence data Shown are results from assignments using (A) RIM-DB, (B) SILVA and (C) Greengenes.

10.7717/peerj.494/supp-6 Table S4 Datasets used to test different databases Sets of partial archaeal 16S rRNA gene sequences (V6–V8) amplified from rumen samples used in this study, together with their accession numbers.

10.7717/peerj.494/supp-7 File S1 Reference database file “RIM_DB_14_6.fasta.zip” This zipped file contains a multi-fasta file (“RIM_DB_14_6.fasta”) that comprises all the sequences in the database, and the three BLAST database files (“RIM_DB_14_6.fasta.nhr”, “RIM_DB_14_6.fasta.nin”, and “RIM_DB_14_6.fasta.nsq”) to use the sequences as references with File S2 or File S3 for BLAST-based assignment of query sequences in QIIME.

10.7717/peerj.494/supp-8 File S2 Taxonomy file “RIM_DB_14_6_f.txt” This taxonomy file allows taxonomic assignment to the isolate level and is recommended for analysis of communities of known composition, such as mock communities. This taxonomy file can be used with File S1 for BLAST-based taxonomic assignments in QIIME.

10.7717/peerj.494/supp-9 File S3 Taxonomy file “RIM_DB_14_6_c.txt” This taxonomy file offers more conservative taxonomic assignments and is recommended for the analysis of environmental sequence data. This taxonomy file can be used with File S1 for BLAST-based taxonomic assignments in QIIME.

10.7717/peerj.494/supp-10 File S4 ARB-database file “RIM_DB_14_6.arb.zip” This is the ARB-readable database file of the sequences comprising RIM-DB. This file also includes the phylogenetic trees shown in the paper in Fig. 1 and in Fig. S1 and Fig. S2.

## Additional Information and Declarations

### DNA Deposition

The following information was supplied regarding the deposition of DNA sequences: The relevant details are listed in Table S4.
