# Supplementary figures and images for: RIM-DB: a taxonomic framework for community structure analysis of methanogenic archaea from the rumen and other intestinal environments
Source: PeerJ. 2014 Aug 5;2:e494. doi: 10.7717/peerj.494 (PMC4137658; doi:10.7717/peerj.494)

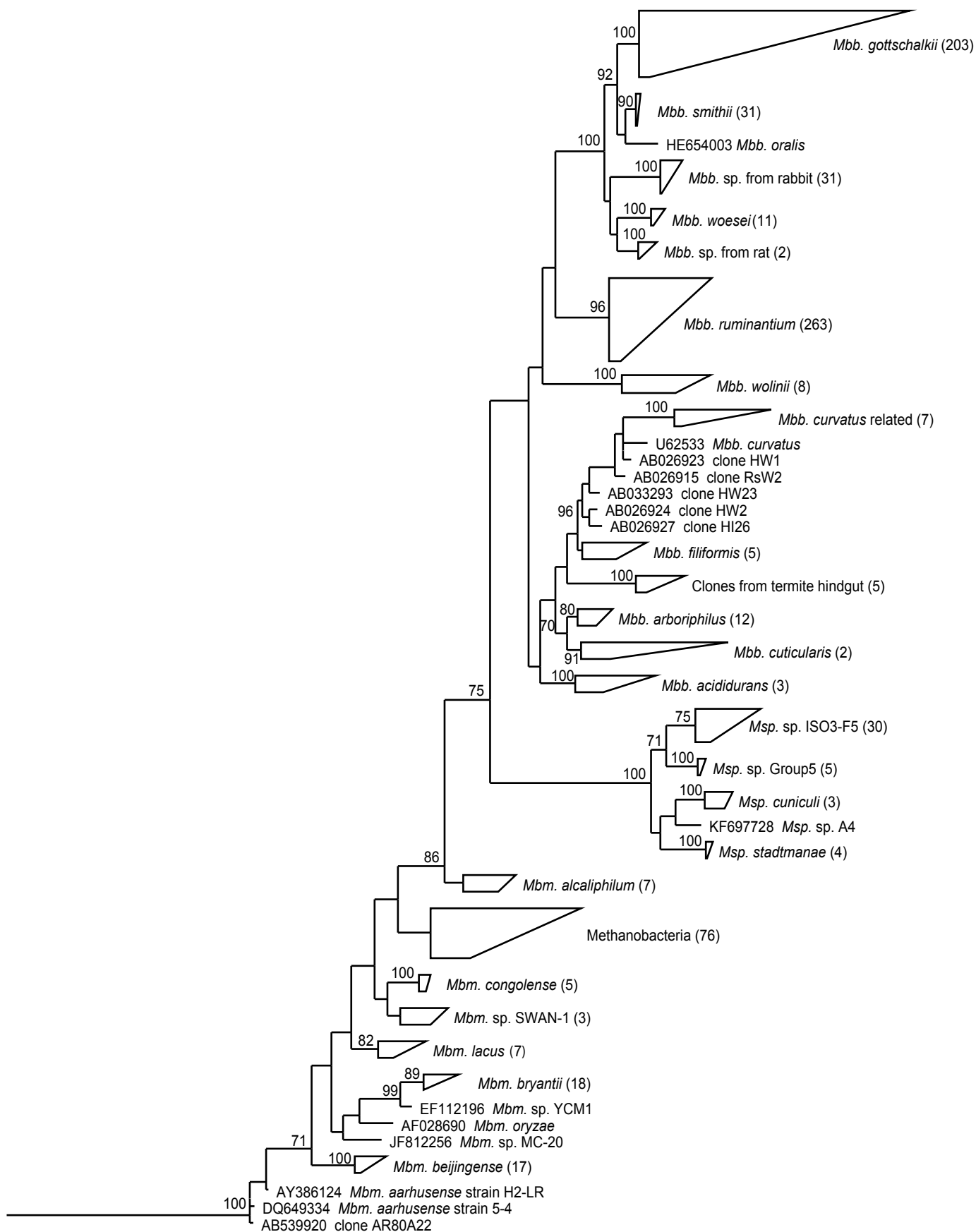

Supplement: Figure S1 — Abbreviations: Mbm. = Methanobacterium, Mbb. = Methanobrevibacter, Msp. = Methanosphaera. The tree was re-sampled 500 times and only bootstrap values ≥70% are shown. The dendrogram was rooted with five Crenarchaeota sequences. The scale bar indicates 0.10 inferred nucleotide substitutions per position. [file peerj-02-494-s001.pdf]

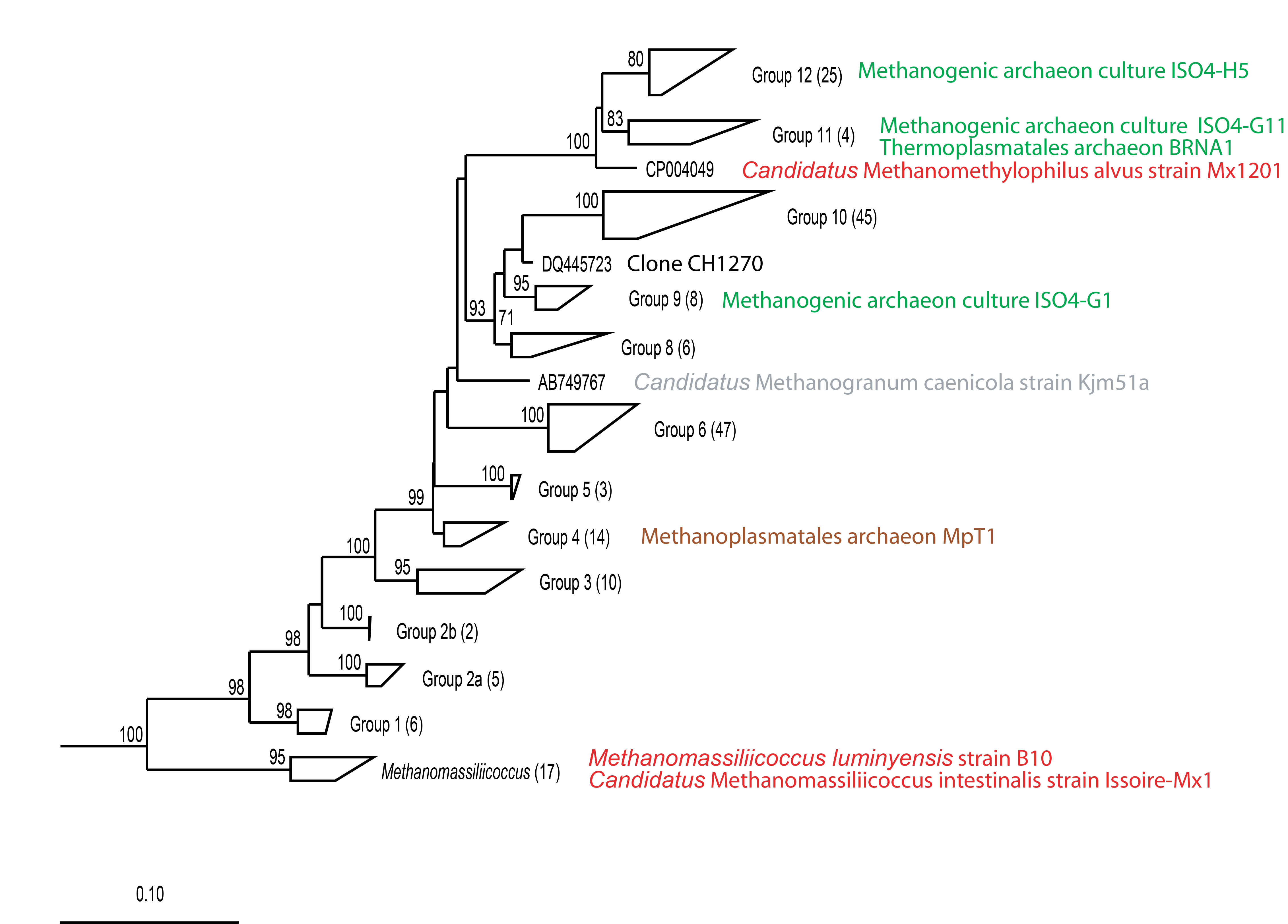

Supplement: Figure S2 — The tree was re-sampled 500 times and only bootstrap values ≥70% are shown. The dendrogram was rooted with 55 sequences from the Archaeoglobales. Available representative isolates/enrichment cultures for the groups are listed after the group name. Alternative group names (Group 2a = TC-2, Group 2b = CC-2, Group 4 contains CC-1, TC-1a, and TC-1b) have been given for some of the groups by Paul and colleagues (2012). The font colour used for the isolate names represents the habitat from which the strains were isolated or enriched: rumen (green), human (red), termite (brown), sludge (grey). Short isolate sequences were not included in the tree. The scale bar indicates 0.10 inferred nucleotide substitutions per position. [file peerj-02-494-s002.png]
